# Endoscopic Sinus Surgery with Antrostomy Has Better Early Endoscopic Recovery in Comparison to the Ostium-Preserving Technique

**DOI:** 10.5402/2012/189383

**Published:** 2012-06-18

**Authors:** Annika Luukkainen, Jyri Myller, Tommi Torkkeli, Markus Rautiainen, Sanna Toppila-Salmi

**Affiliations:** ^1^Department of Otorhinolaryngology, University of Tampere, Finn Medi III, 4th Floor (Biokatu 10), 33520 Tampere, Finland; ^2^Department of Otorhinolaryngology, Paijat-Hame Central Hospital, Keskussairaalankatu 7, 15850 Lahti, Finland; ^3^Department of Otorhinolaryngology, Mikkeli Central Hospital, Porrassalmenkatu 35-37, 50100 Mikkeli, Finland; ^4^Department of Otorhinolaryngology, Tampere University Hospital, PL 2000, Teiskontie 35, 33521 Tampere, Finland; ^5^Department of Allergy, Skin and Allergy Hospital, Helsinki University Central Hospital, P.O. Box 160 (Meilahdentie 2), 00029 Hospital District of Helsinki and Uusimaa, Helsinki, Finland; ^6^Transplantation Laboratory, Haartman Institute, University of Helsinki, FIN-00014 Helsinki, Finland

## Abstract

*Background*. Endoscopic sinus surgery (ESS) is considered for chronic rhinosinusitis (CRS) after failure of conservative therapy. *Objective*. The aim of this study was to evaluate endoscopically ostium patency and mucosal recovery after ESS, with either maxillary sinus ostium-preserving or -enlarging techniques. *Materials and Methods*. Thirty patients with non-polypous CRS were enrolled. Uncinectomy-only and additional middle meatal antrostomy were randomly and single-blindly performed for each side. Pre- and postoperative endoscopic scores were semi-quantitatively determined according to findings in the ostiomeatal complex area. Adhesions, maxillary sinus mucosal swelling, secretions, and ostium obstruction were also endoscopically evaluated. In addition, symptoms were asked and computed tomography scans were taken preoperatively and 9 months postoperatively. *Results*. At 16 days postoperatively, a better endoscopic score and a less obstructed ostium were found with antrosomy. At 9 months postoperatively the endoscopic score improved significantly and identically with both procedures, however, obstructed ostia and sinus mucosal swelling/secretions were insignificantly more frequently found on the uncinectomy-only side. Endoscopic and radiologic findings of the maxillary sinus mucosa and ostium correlated significantly 9 months postoperatively. *Conclusion*. There was a good long-term mucosal recovery with both surgical procedures. In terms of early mucosal recovery and ostium patency, antrostomy might be slighly superior.

## 1. Introduction

Chronic rhinosinusitis (CRS) is an inflammation of the nose and paranasal sinuses lasting more than 12 weeks with a prevalence of about 10% in Europe [1, 2]. It is diagnosed by typical symptoms and/or computed tomography (CT) scan and/or endoscopic changes [1]. After failure of conservative therapy, endoscopic sinus surgery (ESS) aims to restore mucociliary clearance and ventilation through the natural ostia. ESS is based on the theory that the maxillary sinus ostium is the most important area in the pathogenesis of chronic and recurrent rhinosinusitis [3, 4]. Obstruction of the ostium is believed to lead to chronic inflammation and eventually to pathologic alterations of the maxillary sinus mucosa. Therefore, surgical opening of the ostium and thus improved drainage and ventilation of the sinus might restore the normal mucosa [5]. There are different opinions concerning the extent of surgery of the ostiomeatal complex. It is considered that removal of the uncinate process alone would be enough to restore the ventilation of the maxillary sinus. ESS with the minimally invasive technique aims to achieve normal sinus function and prevent sinus exposure to environmental irritants, by causing minimal opening of the sinonasal structures [6, 7]. The effect of minimally invasive ESS has been shown to be comparable to invasive ESS [6, 8–10]. Only few controlled studies have compared small or no widening of bony or cartilaginous structures in the maxillary sinus ostium to antrostomy with relatively promising results [11–15]. On the other hand, uncontrolled studies suggest that the presence of biofilms, osteomyelitis, and other factors favor invasive approaches towards the ostiomeatal unit [16, 17].

Our aim was to compare endoscopically the mucosal recovery of the ostiomeatal complex area and maxillary sinus, after endoscopic sinus surgery with either the ostium—preserving or ostium—enlarging technique.

## 2. Materials and Methods

### 2.1. Subjects

This randomized, single-blinded study was carried out in the Department of Otorhinolaryngology, Tampere University Hospital, Finland, and Mikkeli Central Hospital, Mikkeli, Finland, between 2001 and 2003.

Characteristics of groups of patients can be seen in Table 1. Thirty patients with CRS were enrolled in this study.

Inclusion criteria were moderate-to-severe sinus-related symptoms, according to patient interview,during at least 12 weeks despite maximal medical treatment and a Lund-Mackay (LM) sinus computed tomography (CT) score [18] of at least 6/24 but no more than 18/24.

Exclusion criteria were age less than 18 years; oral corticosteroid treatment during the last two months prior to surgery; previous sinonasal surgery; a history or physical examination suggestive of severe nasal septal deviation (that causes only unilateral nasal obstruction and/or requires septoplasty before ESS can be performed), unilateral sinusitis, nasal polyposis > grade 1 [18], aspirin sensitivity, chronic bronchitis, cystic fibrosis, a tumour or a disease with a severe impact on general immunity.

Dropouts from the study one patient died accidentally prior to the control at 9 months postoperatively.

### 2.2. Sinus Surgery

ESS was performed by two authors Myller et al. as previously described [13, 15, 19]. Briefly, the uncinectomy was performed on both sides, in which the lower two-thirds of the uncinate process was removed. Additional middle meatal antrostomy was randomized on either the right or the left side of each patient. It was performed by removing with cutting forceps, the posterior connective tissue of the natural ostium, to duplicate the diameter. If a large ethmoid bulla was disturbed doing uncinectomy and/or antrostomy, it was opened. The light middle meatal tamponation was removed on the first postoperative day. Nasal endoscopy was performed, and the operation field was cleaned 2 weeks after surgery (Table 1).

### 2.3. Endoscopy

The endoscopic evaluation was performedpreoperatively, 1–77 days (mean ± SD, 26 ± 23 days) before the operation, perioperatively, and during the debridement follow-up visit at 7–30 days (mean ± SD, 16 ± 5 days) postoperatively, 3 and 9 months postoperatively. Physicians filled a form with endoscopic findings. The maxillary sinus ostium obstruction was scored: 0: no and 1: yes. The maxillary mucosa was scored: 0: normal (with or without cyst), 1: edema, and 2: polypous mucosa. The maxillary mucosal sinussecretions were scored: 0: no, 1: mucus, and 2: pus. The endoscopicscore of the ostiomeatal complex area was semiquantitatively determined from the following changes: swollen/polypotic mucosa found in the middle turbinate/anterior ethmoid cells/uncinate process/maxillary sinus ostium/opening of the frontal recess; middle meatal adhesions; anatomical narrowness of the middle meatus. Endoscopic score 0 was normal, 1: mild, 2: moderate, and 3: severe changes of the middle meatus and ostiomeatal complex.

### 2.4. Symptoms

The symptoms were recorded by a questionnaire preoperatively and at 16 days, 3 and 9 months postoperatively. The following symptoms were asked: facial pain/pressure, nasal obstruction, nasal discharge, postnasal drip, decreased sense of smell, and they were scored: no = 0, mild or moderate = 1, and severe = 2. In addition, lacrimation (none = 0, mild = 1, moderate = 2, and severe = 3) and postoperative bleeding (absent = 0, mild or moderate = 1, and severe = 2) were asked during the debridement follow-up visit at 7–30 days (mean ± SD, 16 ± 5 days), and at nine months postoperatively.

### 2.5. Computed Tomography Scans

Coronal sinus CT scans were obtained before and 9 months postoperatively. The ostiomeatal complex was reconstructed with 1 mm slice thickness. LM scores and the area of the ostium were determined, as previously described [13].

### 2.6. Ethical Considerations

The study was approved by the Institutional review boards of the Tampere University Hospital and Mikkeli Central Hospital. All patients suffered from a moderate-severe form of CRS. Informed consent was obtained from all patients.

### 2.7. Statistical Analysis

Statistics were performed with SPSS Base 16.0 Statistical Software Package (SPSS, Chicago, IL, USA). Data are expressed as medians and interquartile ranges. The data was tested and found not to be normally distributed. The nonparametric Wilcoxon rank sum test was used for comparison of matched pairs. Kruskal Wallis and Mann-Whitney *U* tests were used for comparisons of groups. For correlations, the nonparametric Spearman rank correlation test was used. A two-tailed *P* value of less than 0.05 was considered significant in all tests.

## 3. Results

### 3.1. Endoscopic Score

**Figure 1:**
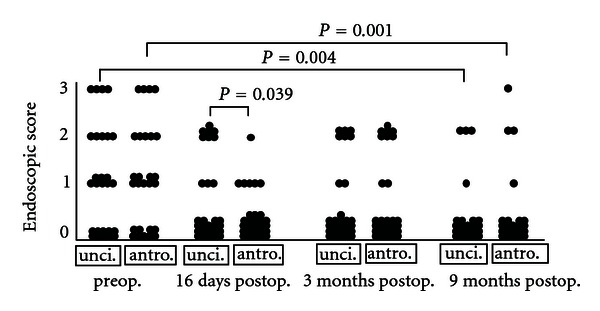
The comparison of endoscopic score between the sides with uncinectomy-only (unci) and additional middle meatal antrostomy (antro) between different time points. The endoscopic score of the middle meatus was semiquantitatively determined: 0: normal, 1: mild, 2: moderate, and 3: severe changes of the middle meatus. *P* values by Wilcoxon test. Only the *P* values < 0.05 are shown in the figure.

**Figure 2:**
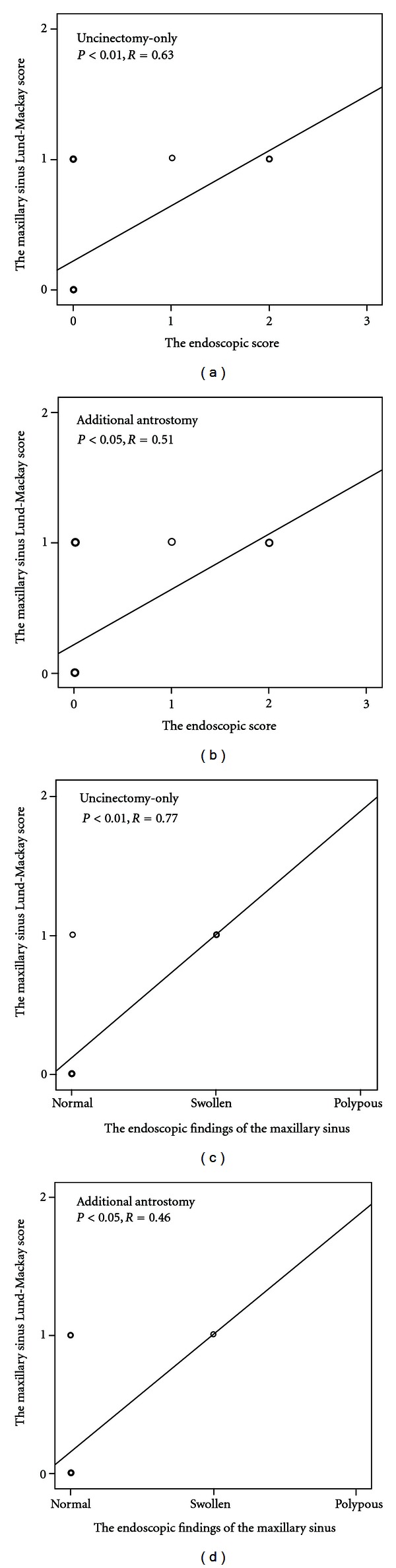
Correlation between maxillary sinus LM-score form CT scans and the endoscopic score of middle meatus on the uncinectomy-only and antrostomy sides at 9 months postoperatively (a) and (b). Correlation between maxillary sinus LM-score and the endoscopic findings of maxillary sinus mucosa on the uncinectomy-only and antrostomy sides at 9 months postoperatively (c) and (d).

We used the endoscopic score to evaluate semiquantitatively the mucosal status of the operated area. A high endoscopic score indicated swollen or polypous mucosa and/or anatomical narrowness of middle meatal and ostiomeatal complex area. Preoperative observation of both sides of each CRS patient revealed no significant differences statistically in the middle meatal endoscopic scores (*P* > 0.05, by Wilcoxon test, Figure 1). The endoscopic scores did not change significantly between the preoperative, and 16-day and 3-month postoperative periods (*P* > 0.05, by Wilcoxon test, Figure 1). Nor did it change significantly between the 16-day and 9-month periods (*P* > 0.05, by Wilcoxon test, Figure 1). However, when comparing the preoperative endoscopy scores to 9-month postoperative endoscopic scores of each side separately, a significant and identical improvement on both the ostium preserving and enlarging sides was observed (*P* = 0.004, *P* = 0.001, resp., by Wilcoxon test, Figure 1). The endoscopic score was better on the antrostomy side compared to the uncinectomy-only side, but only at 16 days postoperatively (*P* = 0.039, by Wilcoxon test, Figure 1). Interestingly, at 9 months postoperatively there was a correlation between the endoscopic score and the radiologic maxillary sinus LM score (the uncinectomy-only side *P* < 0.01, *R* = 0.63; the antrostomy side *P* < 0.05, *R* = 0.51, by Spearman rank correlation test, Figures 2(a) and 2(b)). Preoperatively, there were no correlations between these variables (*P* < 0.05 by Spearman rank correlation test, data not shown). Allergic rhinitis associated with a higher endoscopic middle meatal score, but only on the ostium-enlarging side and at 9 months postoperatively (*P* = 0.037 by Mann-Whitney *U* test, data not shown). In contrast, the endoscopic score did not associate with age, sex, smoking, asthma, or medication (*P* > 0.05, by Mann-whitney *U* and Spearman rank correlation tests, data not shown). Nor did the pre- or post-operative endoscopic scores correlate with any of the symptoms asked at the same time points on either ostium—preserving or-enlarging sides (*P* > 0.05 by Spearman rank correlation test, data not shown).

### 3.2. Maxillary Sinus Ostium

There were no peri- or post-operative differences between the operation techniques in terms of accessory ostium, endoscopically evaluated (*P* > 0.05, by Wilcoxon test, data not shown). At 16 days post-operatively, eight obstructed maxillary sinus ostia were found on the uncinectomy-only side in contrast to only one on the antrostomy side (*P* = 0.031, by Wilcoxon test, Table 2). Of these, five remained obstructed at also 3 and/or 9 months postoperatively, four on the uncinectomy-only side and one on the antrostomy side (Table 2). At 9 months postoperatively two new ostium obstructions were identified with each technique leading to identical numbers of obstructed ostia between the sides (*P* > 0.05 by Wilcoxon test, Table 2). In one case, the ostium was seen as being obstructed endoscopically but turned out to be widely open on CT-scans taken 9 months postoperatively (Table 2). When dropping out this exceptional case, the ostium findings by CT-scans and by endoscopy were otherwise in line at 9 months postoperatively: the median of radiologic ostium area was higher in cases with endoscopically patent ostium contrasted to those with endoscopically obstructed ostium (the uncinectomy-only side *P* = 0.003, the antrostomy side *P* = 0.009, by Mann-Whitney *U* test, data not shown). Sex, allergic rhinitis, asthma, smoking, nasal corticosteroids and/or antihistamine use did not associate with an obstructed maxillary sinus ostium (*P* > 0.05, by Fisher test, data not shown). Nor did maxillary sinus obstruction associate to any of the asked symptoms at 9 months postoperatively (*P* > 0.05, by Mann-Whitney *U* test, data not shown).

### 3.3. Maxillary Sinus Mucosa

There were no perioperative differences between the sides in terms of maxillary mucosal edema and secretions, endoscopically evaluated (*P* > 0.05, by Wilcoxon test, data not shown).At 9 months postoperatively, there was an insignificant trend that maxillary sinus mucosal edema was more frequently found on the uncinectomy-only side compared to the antrostomy side (*P* = 0.083, by Wilcoxon test, Table 2). Furthermore, at 9 months postoperatively four patients had mucous secretions in the maxillary sinus (Table 2). Although three of these were found on the uncinectomy-side, there were no statistically significant differences between the procedures (*P* > 0.05, by Wilcoxon test, Table 2). None of the patients had pus or polypous mucosa in the maxillary sinus. Endoscopic findings ofmaxillary secretions did not associate with maxillary mucosal edema on either antrostomy or uncinectomy-only sides compared separately (*P* > 0.05, by Fisher test, data not shown). However, there was a correlation between maxillary sinus secretions and endoscopic middle meatal score at 9 months postoperatively (the uncinectomy-only side *P* < 0.01, *R* = 0.67; the antrostomy side *P* < 0.01, *R* = 0.58, by Spearman rank correlation test, data not shown). At nine months postoperatively, endoscopically evaluated maxillary mucosal edema correlated with the radiologic maxillary sinus LM score on both uncinectomy and antrostomy sides (*P* < 0.01, *R* = 0.77; *P* < 0.05, *R* = 0.46, resp., by Spearman rank correlation test, Figures 2(c) and 2(d)). In contrast, a swollen maxillary sinus mucosa or secretions did not associate with any of the symptoms asked postoperatively at the same time points on either ostium-preserving or-enlarging sides (*P* < 0.05, by Spearman rank correlation test, data not shown). Nor did a swollen maxillary sinus mucosa or secretions associate to age, sex, allergic rhinitis, smoking, asthma, or medication (*P* > 0.05, by Kruskal-Wallis, Mann-Whitney *U*, and Spearman rank correlation tests, data not shown).

### 3.4. Adhesion Formation

Preoperatively, there were no signs of adhesions. At 9 months postoperatively, six patients out of 29 had endoscopic findings of adhesion formation; three were on the uncinectomy side and four on the antrostomy side (Table 2). There were no differences between sides in terms of adhesions (*P* > 0.05, by Wilcoxon test, data not shown). Adhesion formation did not associate to sex, allergic rhinitis, smoking, nasal corticosteroids, and/or antihistamine (*P* > 0.05, by Fisher test, data partly shown in Table 2). Nor did the presence of adhesions associate to any of the asked symptoms at 9 months postoperatively (*P* > 0.05, by Mann-Whitney *U* test, data not shown).

## 4. Discussion

The important postoperative endoscopic signs are ostium patency, mucosal recovery, and adhesion formation. We found that the side with additional antrostomy showed significantly better early recovery of the middle meatal mucosa and maxillary sinus ostium patency. During nine-month followup, maxillary sinus ostium obstruction was insignificantly more frequently found with the ostium-preserving technique. So were maxillary sinus mucosal swelling and secretions. On the other hand, long-term recovery of the middle meatal mucosa was statistically similarly achieved with both ostium-preserving and ostium-enlarging procedures. Similarly to our findings, Wadwongtham and Aeumjaturapat. showed in a randomized study that antrostomy was better than uncinectomy early postoperatively, but that at one year postoperatively a 60% rate of patency was achieved with both procedures [14]. One study comparing the antrostomy size, as well as other observational studies, showed good endoscopic recovery after ESS [20, 21].

Our finding that half of early postoperatively obstructed ostia remained obstructed later on with other signs of poor recovery seems to be in accordance with a recent study showing that findings of good recovery even at one month postoperatively seem to predict good long-term results of ESS [22]. On the other hand, previous observations have shown that postoperative mucosal healing takes more than one month [23–26]. Endoscopic parameters, such as middle turbinate position, adhesions, inflammation, and crusting, have shown to have acceptable interexaminer reproducibility and are suitable for evaluating ESS outcomes in the postsurgical period [27, 28].

About two-thirds of patients with recalcitrant CRS might have biofilms in the sinonasal mucosa, but their influence on disease or ESS outcomes have yet to be elucidated [11, 16, 17]. Zhang et al. have observed that bacterial biofilms might associate to asthma and also to adhesion formation and revision ESS [29]. In case of revision sinus surgery, Kennedy has argued in favour of complete uncinate process removal, whilst preserving the mucosa [30]. Wang et al. showed a statistically significant correlation between more advanced bone remodelling and a higher postoperative endoscopic score, thus also reinforcing the putative importance of operating diseased bony/cartilage structures in order to achieve mucosal recovery [31]. In our study, it seems that antrostomy as a more invasive procedure does not cause more frequently adhesion formation.

Patient history factors failed to provide an explanation for the development of adhesions, maxillary ostium obstruction, or mucosal findings. The only exception was allergic rhinitis, which associated with a higher endoscopic middle meatal score, which is not easy to explain as we did not observe increased inferior turbinate swelling in atopic patients pre- or post-operatively [32]. In contrast to what has been found in patients with Samter's triad, allergic rhinitis would not seem to affect CRS severity or outcomes of ESS, as long as atopy is taken into account and treated [33–37]. Tomassen et al. have shown that most patients with CRS symptoms, and also about a third of those without CRS symptoms, have a positive nasal endoscopy [38]. Endoscopic findings did not associate with symptoms in our study, which is in accordance with other observations [20, 39, 40].

 The present and previous studies of ours and others show that nasal endoscopy findings correlate strongly with CT-scan scores, thus arguing in favour of radiation reduction by performing proper endoscopy [20, 38, 39]. Still, both CT imaging and endoscopy might be needed, as was shown in a study where CT scans were superior in detecting anatomical variations in all bony or cartilaginous structures, while endoscopy remained superior only in polyp diagnostics [40].

As a methodological shortcoming, with this study setup we were not able to observe the influence of comorbidities on procedure outcomes. On the other hand, the patient was his or her own control which decreases the effect of confounding factors, such as interpatient differences in use of pre-or post-operative medication and early postoperative care.

## 5. Conclusion

Overall, there was a good and similar long-term endoscopic recovery of the middle meatal and ostiomeatal complex area after maxillary sinus surgery with either the ostium-preserving or-enlarging technique. Antrostomy is, however, better in early middle meatal recovery and ostium patency. This might putatively influence long-term results also. Postoperatively, endoscopy and CT scans provide identical information about the ostiomeatal complex area and maxillary sinus.

## Figures and Tables

**Table 1:** 

Characteristics of patients	Preoperative	Postoperative 9 months
	*n* = 30	*n* = 29
Age, years:		
median	50	
range	21–66	
Number of males (%)	10 (33.3)	10 (34.5)
Number of patients with allergic rhinitis (%)	17 (56.7)	17 (58.6)
Number of patients with asthma (%)	10 (33.3)	10 (34.5)
Number of patients with nasal polyps (%)	0	1 (3.4)
Smokers (%)	8 (26.7)	7 (24.1)
Number of patients without opening of the ethmoid bulla on both sides (%)	5 (16.7)	4 (13.8)
No. of patients using:		
Antihistamine (%)	2 (6.7)	1 (3.4)
intranasal corticosteroids (%)	8 (26.7)	6 (20.7)
Both (%)	4 (13.3)	3 (10.3)

**Table 2:** The patients with endoscopic signs of ostium obstruction and/or adhesion formation as complications. Both pre- and post-operative endoscopic scores (0: normal, 1: mild, 2: moderate, and 3: severe changes of middle meatus) are shown. Other endoscopic findings −: no; +: yes; adhesions: ++++: ostium severely restricted, +++: from lateral wall of middle meatus to middle turbinate, ++: from middle turbinate to septum, +: from lower turbinate to septum; ^1^patients with missing data, thus the 3-month postoper. data was used, +^2^: the ostium was widely open in sinus CT scans 9 months postoperatively; ?: the maxillary sinus mucosa was not seen; M: male, F: female; d.: days; m.: months. None of the patients suffered from any acute infection, nor had endoscopic signs of polypous maxillary sinus mucosa or pus secretions in maxillary sinus.

Gender		M^1^	M^1^	M	M	M	F^1^	F	F	F	F	F	F	F
Age		46	53	59	63	31	60	22	50	31	49	23	40	65
Allergic rhinitis		+	−	−	−	−	−	+	+	+	+	−	−	−
Asthma		−	−	+	+	+	−	−	−	+	+	−	−	−
Use of intraasal corticosteroids		−	−	−	+	−	−	−	−	+	−	−	+	−
Smoking		−	+	+	−	−	+	−	−	−	+	+	−	−

Maxillary sinus ostium obstruction														
16 d. postop.	Uncinectomy	+	+	+	+	−	+	−	−	−	+	+	+	−
9 m. postop.	Uncinectomy	−	+	+	−	+	−	−	+	−	−	+	+	−
16 d. postop.	Antrostomy	−	−	−	+	−	−	−	−	−	−	−	−	−
9 m. postop.	Antrostomy	+	−	+^2^	+	+	−	−	−	−	+	−	−	−
Adhesions														
9 m. postop.	Uncinectomy	+++	−	−	−	+++	+	+++	−	−	−	+++	−	−
9 m. postop.	Antrostomy	−	−	+++	−	+++	−	−	−	+	−	−	−	+++

Endoscopic score of the middle meatus														
Preoperative	Uncinectomy	0	0	3	0	2	0	1	1	0	3	1	1	1
9 m. postop.	Uncinectomy	0	2	0	1	0	0	0	2	0	0	2	2	0
Preoperative	Antrostomy	0	2	3	0	2	0	1	1	0	3	1	1	1
9 m. postop.	Antrostomy	2	0	3	2	2	0	0	0	0	2	1	0	0

Swollen maxillary sinus mucosa														
9 m. postop.	Uncinectomy	−	−	?	+	−	−	−	+	+	?	−	+	−
9 m. postop.	Antrostomy	−	−	?	+	−	−	−	−	−	?	−	−	−

Mucus secretions of maxillary sinus														
9 m. postop.	Uncinectomy	−	−	−	−	+	−	−	+	+	−	−	−	−
9 m. postop.	Antrostomy	−	−	−	−	+	−	−	−	−	−	−	−	−
